# Author Correction: Rational engineering of an elevator-type metal transporter ZIP8 reveals a conditional selectivity filter critically involved in determining substrate specificity

**DOI:** 10.1038/s42003-023-05460-3

**Published:** 2023-10-24

**Authors:** Yuhan Jiang, Zhen Li, Dexin Sui, Gaurav Sharma, Tianqi Wang, Keith MacRenaris, Hideki Takahashi, Kenneth Merz, Jian Hu

**Affiliations:** 1 Department of Chemistry, Michigan State University, East Lansing, MI 48824 USA; 2 Department of Biochemistry and Molecular Biology, Michigan State University, East Lansing, MI 48824 USA; 3 Department of Microbiology & Molecular Genetics, Michigan State University, East Lansing, MI 48824 USA

**Keywords:** Ion transport, Transporters

Correction to: *Communications Biology* 10.1038/s42003-023-05146-w, published online 26 July 2023.

In this article, the grant number NIH GM135018, GM115848 & GM038784 relating to Department of Microbiology & Molecular Genetics, Michigan State University, East Lansing, MI 48824, USA. for Keith MacRenaris was omitted. The original article has been corrected.

