# Supplementary material for: Rational engineering of an elevator-type metal transporter ZIP8 reveals a conditional selectivity filter critically involved in determining substrate specificity
Source: Commun Biol. 2023 Jul 26;6:778. doi: 10.1038/s42003-023-05146-w (PMC10372143; doi:10.1038/s42003-023-05146-w)
Supplement: Supplementary file 2 — Supplementary Information [file 42003_2023_5146_MOESM2_ESM.pdf]

Supplementary information

Rational engineering of an elevator-type metal transporter ZIP8 reveals a conditional selectivity filter critically involved in determining substrate specificity

Yuhan Jiang[1], Zhen Li[1], Dexin Sui[2], Gaurav Sharma[1], Tianqi Wang[2], Keith MacRenaris[3], Hideki Takahashi[2], Kenneth Merz[1,2], Jian Hu[1,2,#]

[1]Department of Chemistry, Michigan State University, East Lansing, MI 48824, United States

[2]Department of Biochemistry and Molecular Biology, Michigan State University, East Lansing, MI 48824, United States

[3]Department of Microbiology & Molecular Genetics, Michigan State University, East Lansing, MI 48824, United States

[#]To whom correspondence should be addressed: Jian Hu, hujian1@msu.edu

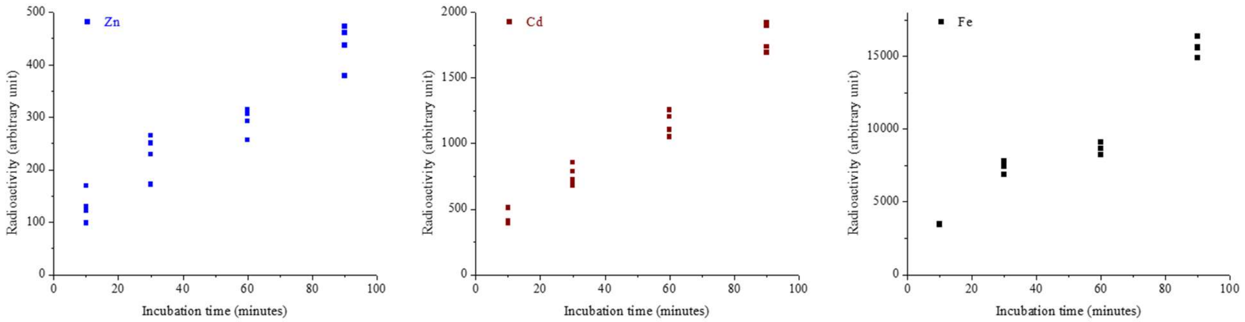

Supplementary Figure 1. Time courses of metal transport by the wild type ZIP8. *left*: Zn^{2+}; m*iddle*: Cd^{2+}; *right*: Fe^{2+}. Metal substrates were detected using a gamma counter (for ^{65}Zn and ^{109}Cd) or a liquid scintillation counter (for ^{55}Fe). The concentrations of Zn^{2+}, Cd^{2+}, and Fe^{2+} were 5 μM, 5 μM, and 20 μM respectively. The shown data are from one representative experiment of three independent experiments with three replicates for each condition.

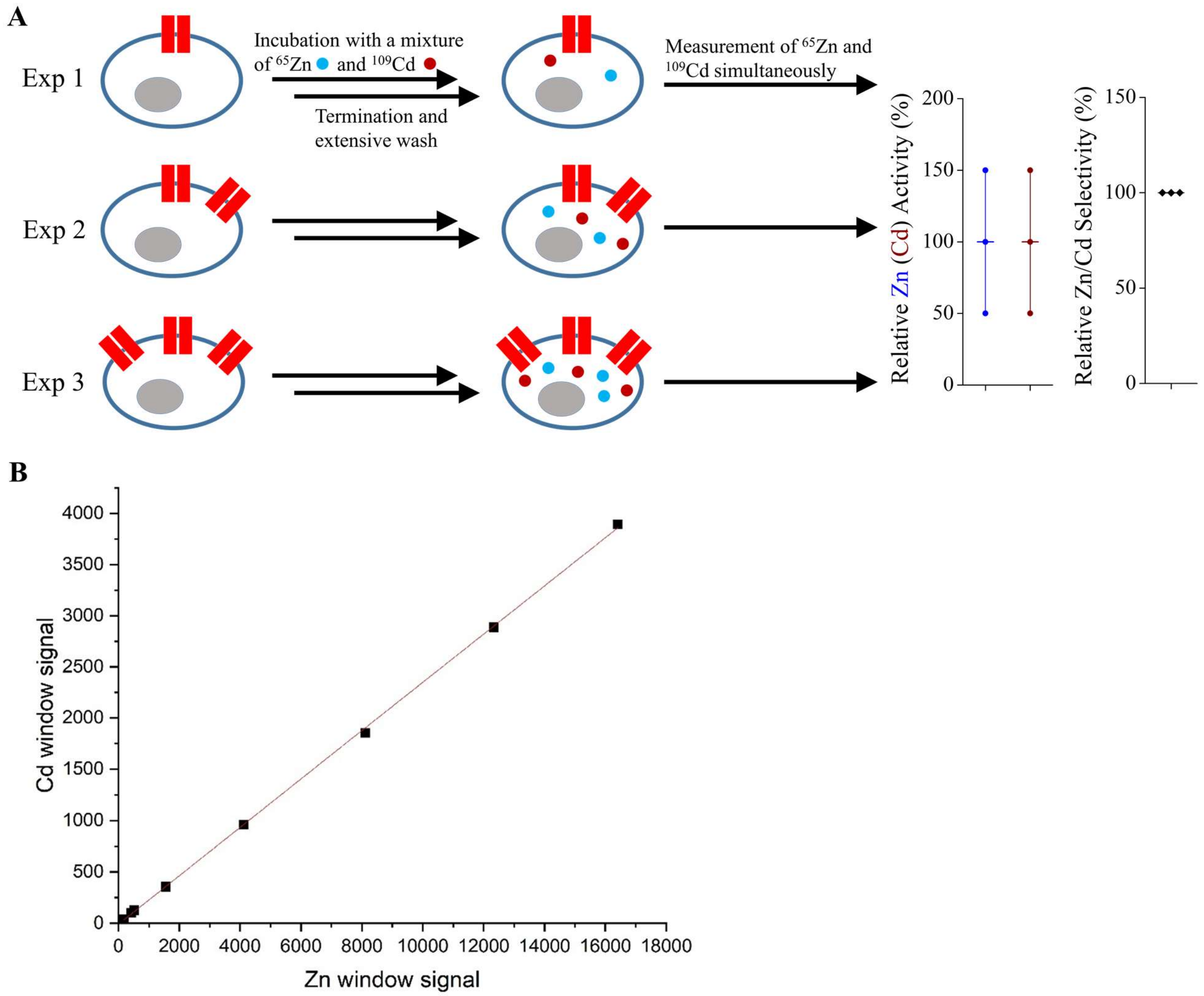

Supplementary Figure 2. Internal competition transport assay and data processing. (**A**) Illustration of the experimental procedure. Cells expressing different levels of ZIP8 (shown as red channels) in three independent experiments (Exp 1-3) are incubated with a mixture of ^{65}Zn and ^{109}Cd under the same experimental conditions, followed by termination with an ice cold EDTA-containing solution and extensive wash. Radioactivities of ^{65}Zn and ^{109}Cd associated with the cells were simultaneously quantified by using a gamma counter in two detection windows (800-1500keV for Zn and 30-150keV for Cd). As demonstrated in the simulated experiments, although the Zn (or Cd) transport activity may have a large deviation due to varied expression levels of the transporter in different experiments, the ratios of the transport activities of Zn to Cd (the Zn/Cd selectivity) are essentially the same. (**B**) Calibration of the signals recorded in the Cd window. Radioactivities of a series of ^{65}Zn standard samples (0-20 μM) recorded in the Cd window are plotted against the readings recorded in the Zn window. The slope of the curve (0.235) means that, for any given sample containing ^{65}Zn and ^{109}Cd, 23.5% of the reading recorded in the Zn window contributes to the reading in the Cd-window. Therefore, to calibrate the readings in the Cd window, 23.5% of the reading in the Zn window was subtracted from the reading recorded in the Cd window to determine the radioactivity truly derived from ^{109}Cd. ^{109}Cd does not contribute to the reading in the Zn window.

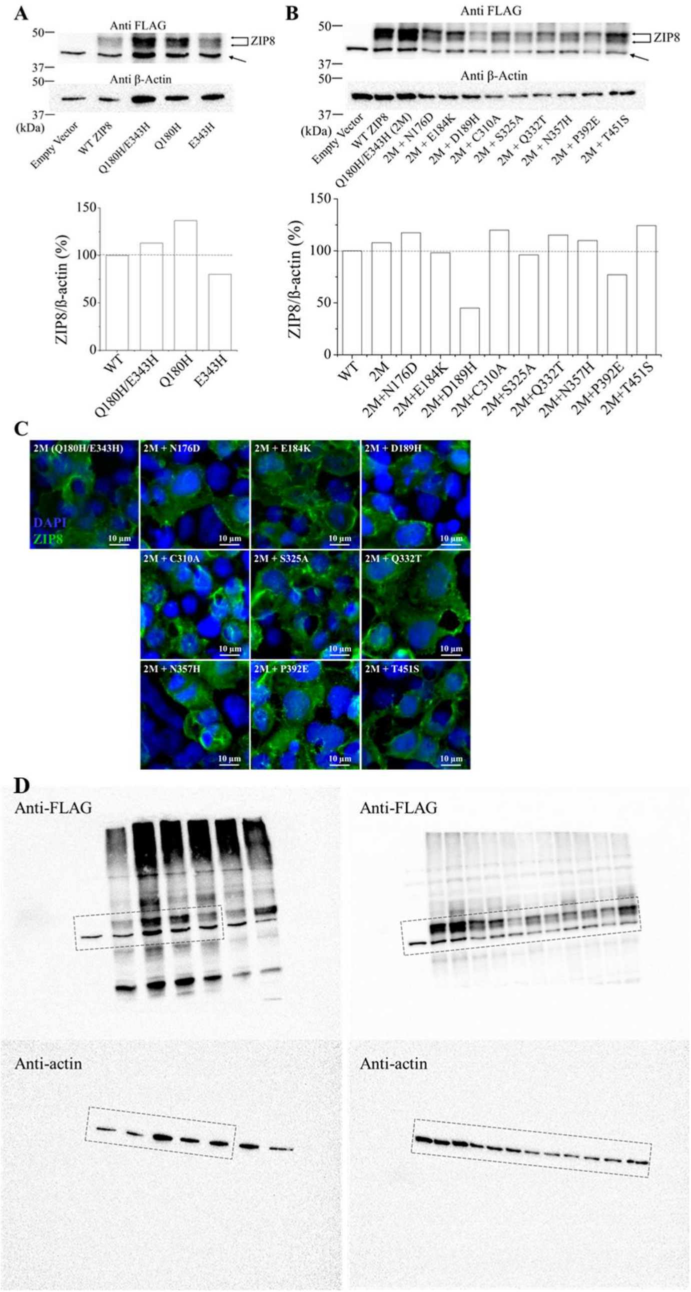

Supplementary Figure 3. Expression analysis of ZIP8 and the variants. (**A**) Comparison of the single and double variants with the wild type ZIP8 by Western blot (upper panel) and quantification analysis using ImageJ (lower panel). (**B**) Comparison of triple variants with the wild type ZIP8 and the Q180H/E343H (2M) variant by Western blot (upper panel) and quantification analysis using ImageJ (lower panel). N-FLAG ZIP8 and β-actin were detected using anti-FLAG and anti-actin antibodies, respectively. The expression level of a variant is expressed as the percentage of the ratio of the wild type ZIP8 over β-actin. The shown result is from a representative experiment. (**C**) Immunofluorescence analysis of cell surface expressed ZIP8 variants. The procedure is the same as indicated in the legend of Figure 4A. (**D**) Uncropped Western blots. The lanes in the dashed frame are shown in (A) and (B).

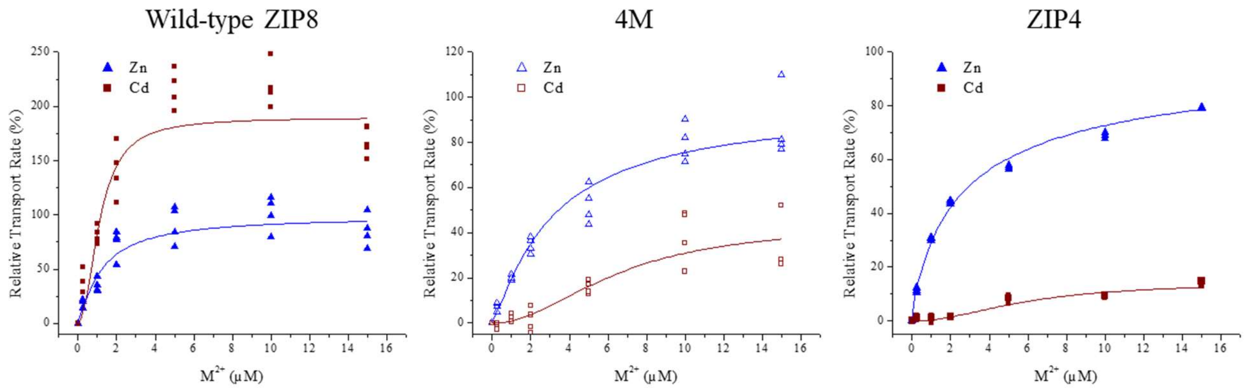

Supplementary Figure 4. The Zn and Cd transport activities of the wild type ZIP8 and the 4M variant (*left*) in comparison with human ZIP4 (*right*). The same data shown in Figure 3B are presented differently for better comparison with ZIP4. The transport rates are expressed as the percentages of the V_{max} for Zn^{2+}. The shown data are from one representative experiment of three independent experiments with three replicates for each condition.

A

Metal ion	Imidazole (His)			Acetate (Asp and Glu)		
	logK	Energy (kcal/mol)	Polarizability on coordinating nitrogen	logK	Energy (kcal/mol)	Polarizability on coordinating oxygen
Zn^{2+}	2.55	-3.48	2.71	1.58	-2.16	0.77
Fe^{2+}	1.80	-2.46	N/A	1.40	-1.91	N/A
Mn^{2+}	1.20	-1.64	N/A	1.40	-1.91	N/A
Cd^{2+}	2.66	-3.63	3.20	1.93	-2.63	1.17

B

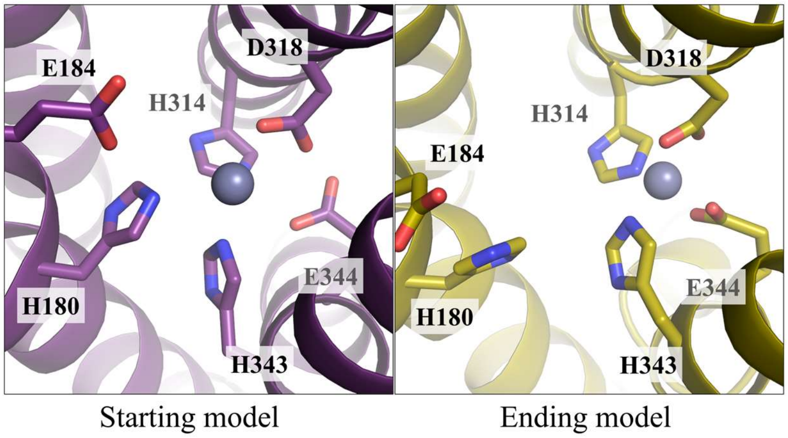

Supplementary Figure 5. Computational characterization of metal binding at the selectivity filter. (**A**) Free energy changes of metal ion binding with small molecule ligands (refs 41&42). (**B**) The initial trial of MD simulation. The zinc ion initially located at the selectivity filter (*left*) moved to the transport site in the ending model (*right*) where it is coordinated with the residues from M1 (H314, H343), M2 (E344), and a bridging residue involved in both M1 and M2 (D318).

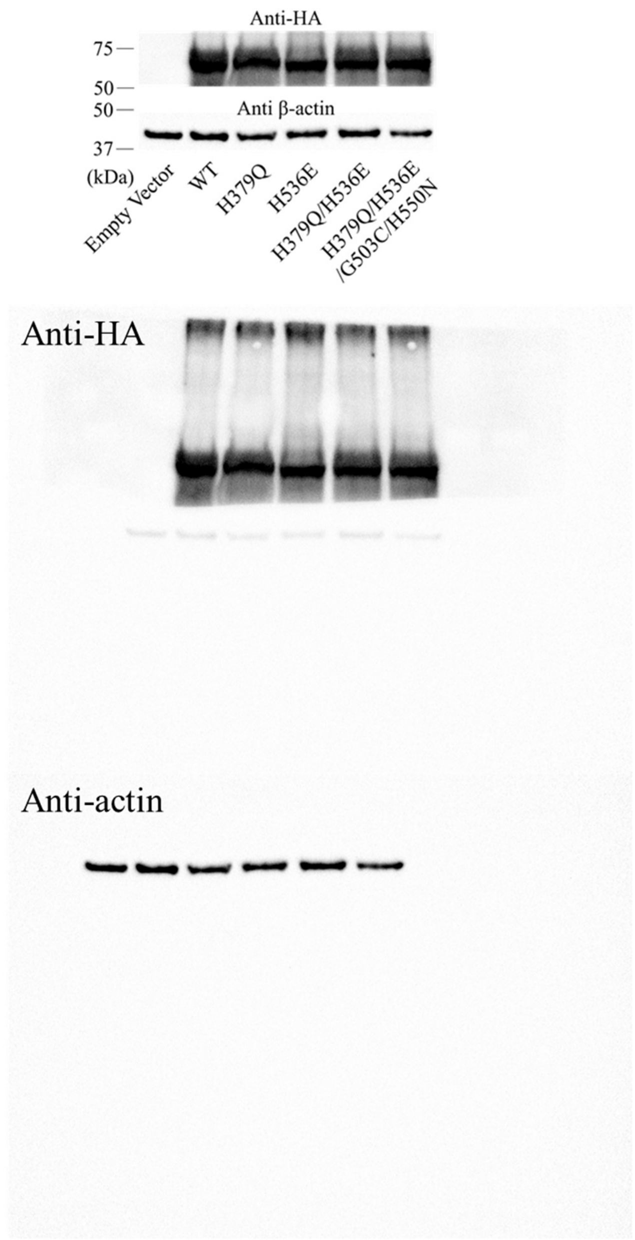

Supplementary Figure 6. Expression analysis of ZIP4 and the variants by Western blot. Human ZIP4 with a C-terminal HA tag and β-actin were detected using anti-HA and anti-β-actin antibodies, respectively. The uncropped Western blots are also shown.

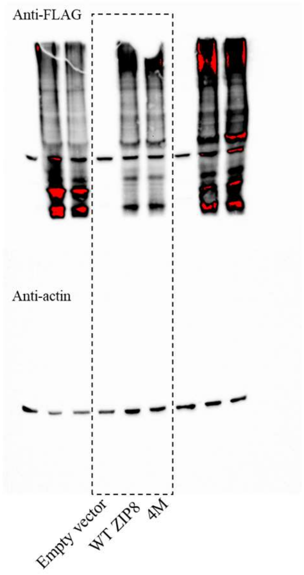

Supplementary Figure 7. Uncropped Western blots of ZIP8 and the 4M variant. The lanes in the dashed frame are shown in Figure 4A.

Supplementary Table 1. Sequence identity of the human LIV-1 proteins

(%)	ZIP7	ZIP13	ZIP4	ZIP12	ZIP8	ZIP14	ZIP5	ZIP6	ZIP10
ZIP7	100	35	27	23	22	24	28	25	27
ZIP13	35	100	29	24	21	24	29	23	23
ZIP4	27	29	100	32	31	30	31	26	25
ZIP12	23	24	32	100	29	31	26	25	27
ZIP8	22	21	31	29	100	48	27	31	30
ZIP14	24	24	30	31	48	100	25	30	29
ZIP5	28	29	31	26	27	25	100	32	35
ZIP6	25	23	26	25	31	30	32	100	40
ZIP10	27	23	25	27	30	29	35	40	100

Supplementary Table 2. The primers for mutagenesis.

Name	Sequence (5'-3') [a]
**ZIP8**	
E343H	ACTTCCATAGCAATCCTATGTCACGAGTTTCCCCACGAGTTAGGA
C374L	CTATTCAACTTCCTTTCTGCATTGTCCTGCTATGTTGGGCTAGCT
C374L/C376A	CTATTCAACTTCCTTTCTGCATTGTCCGCTTATGTTGGGCTAGCTTTTGGC
Q180H	CTTTTTTCAAATGCAATTTTCCACCTTATTCCAGAGGCATTTGGA
Q180H/E343H/N176D	GCTATTGGGACTCTTTTTTCAGATGCAATTTTCCACCTTATTCCA
Q180H/E343H/E184K	GCAATTTTCCACCTTATTCCAAAGGCATTTGGATTTGATCCCAAA
Q180H/E343H/D189H	ATTCCAGAGGCATTTGGATTTCACCCCAAAGTCGACAGTTATGTT
Q180H/E343H/C310A	ATTGCCTGGATGATAACGCTCGCTGATGCCCTCCACAATTTCATC
Q180H/E343H/S325A	GATGGCCTGGCGATTGGGGCTGCTTGCACCTTGTCTCTCCTTCAG
Q180H/E343H/Q332T	TCCTGCACCTTGTCTCTCCTTACAGGACTCAGTACTTCCATAGCA
Q180H/E343H/N357H	GGAGACTTTGTGATCCTACTCCACGCAGGGATGAGCACTCGACAA
Q180H/E343H/P392E	TTGGTGGGCAACAATTTCGCTGAGAATATTATATTTGCACTTGCT
Q180H/E343H//T451S	ACTGGAAGAAAAACCGATTTCTCATTCTTCATGATTCAGAATGCT
**ZIP4**	
H379Q	CTCACTGGGGACGCTGTCCTGCAACTGACGCCCAAGGTGCTGGGG
H536E	ACCTCGCTGGCCGTGTTCTGCGAAGAGTTGCCACACGAGCTGGGG
G503C	CTGCCCTATATGATCACTCTGTGTGACGCCGTGCACAACTTCGCC
H550N	GGGGACTTCGCCGCCTTGCTGAACGCGGGGCTGTCCGTGCGCCAA

[a] Only the forward primers are shown. The reverse primers are reversely complimentary to the sequences of the forward primers.

[b] The Q180H/E343H/C310A/N357H variant was generated by using the primers for the Q180H/E343H/C310A and N357H variants.

Supplementary Table 3. ICP-MS analysis of some *d*-block metals in the culture media (DMEM+10% FBS) before and after the treatment with the Chelex-100 resin.

(μM)	Mn	Fe	Zn
Before	0.059	3.988	3.637
After	0.043	3.702	0.091
