# Supplementary material for: Rational engineering of an elevator-type metal transporter ZIP8 reveals a conditional selectivity filter critically involved in determining substrate specificity
Source: Commun Biol. 2023 Jul 26;6:778. doi: 10.1038/s42003-023-05146-w (PMC10372143; doi:10.1038/s42003-023-05146-w)
Supplement: Supplementary file 5 — Reporting Summary [file 42003_2023_5146_MOESM5_ESM.pdf]

Corresponding author(s): Jian Hu

Last updated by author(s): Jul 8, 2023

Reporting Summary

Nature Portfolio wishes to improve the reproducibility of the work that we publish. This form provides structure for consistency and transparency in reporting. For further information on Nature Portfolio policies, see our Editorial Policies and the Editorial Policy Checklist.

Statistics

For all statistical analyses, confirm that the following items are present in the figure legend, table legend, main text, or Methods section.

n/a	Confirmed	
☐	☒	The exact sample size (*n*) for each experimental group/condition, given as a discrete number and unit of measurement
☐	☒	A statement on whether measurements were taken from distinct samples or whether the same sample was measured repeatedly
☐	☒	The statistical test(s) used AND whether they are one- or two-sided<br>*Only common tests should be described solely by name; describe more complex techniques in the Methods section.*
☒	☐	A description of all covariates tested
☒	☐	A description of any assumptions or corrections, such as tests of normality and adjustment for multiple comparisons
☐	☒	A full description of the statistical parameters including central tendency (e.g. means) or other basic estimates (e.g. regression coefficient) AND variation (e.g. standard deviation) or associated estimates of uncertainty (e.g. confidence intervals)
☐	☒	For null hypothesis testing, the test statistic (e.g. *F*, *t*, *r*) with confidence intervals, effect sizes, degrees of freedom and *P* value noted<br>*Give P values as exact values whenever suitable.*
☒	☐	For Bayesian analysis, information on the choice of priors and Markov chain Monte Carlo settings
☒	☐	For hierarchical and complex designs, identification of the appropriate level for tests and full reporting of outcomes
☒	☐	Estimates of effect sizes (e.g. Cohen's *d*, Pearson's *r*), indicating how they were calculated

Our web collection on statistics for biologists contains articles on many of the points above.

Software and code

Policy information about availability of computer code

Data collection	N/A
Data analysis	OriginPro8

For manuscripts utilizing custom algorithms or software that are central to the research but not yet described in published literature, software must be made available to editors and reviewers. We strongly encourage code deposition in a community repository (e.g. GitHub). See the Nature Portfolio guidelines for submitting code & software for further information.

Data

Policy information about availability of data

All manuscripts must include a data availability statement. This statement should provide the following information, where applicable:

- Accession codes, unique identifiers, or web links for publicly available datasets
- A description of any restrictions on data availability
- For clinical datasets or third party data, please ensure that the statement adheres to our policy

The data that support this study are available from the corresponding authors upon reasonable request. The structural data cited in this study are available under accession codes 5TSB in PDB. The AlphaFold predicted human ZIP13 structure is retrieved from https://alphafold.ebi.ac.uk/entry/Q96H72. The source data underlying Figures 1, 3, 4, 5, 8 are provided as a Source Data file, which is provided with this paper.

Research involving human participants, their data, or biological material

Policy information about studies with human participants or human data. See also policy information about sex, gender (identity/presentation), and sexual orientation and race, ethnicity and racism.

Reporting on sex and gender	No information was collected.
Reporting on race, ethnicity, or other socially relevant groupings	No information was collected.
Population characteristics	No information was collected.
Recruitment	No information was collected.
Ethics oversight	No information was collected.

Note that full information on the approval of the study protocol must also be provided in the manuscript.

Field-specific reporting

Please select the one below that is the best fit for your research. If you are not sure, read the appropriate sections before making your selection.

☒ Life sciences ☐ Behavioural & social sciences ☐ Ecological, evolutionary & environmental sciences

For a reference copy of the document with all sections, see nature.com/documents/nr-reporting-summary-flat.pdf

Life sciences study design

All studies must disclose on these points even when the disclosure is negative.

Sample size	Three replicates for each condition in one independent experiment. Three or more independent experiments were conducted for each condition.
Data exclusions	No data were excluded.
Replication	Three replicates for each condition in one independent experiment. All the results from at least three independent experiments were used for analysis.
Randomization	Not relevant to this work since no individual samples were used in this study.
Blinding	Not relevant to this work. The people doing experiments knew exactly what they were working on.

Reporting for specific materials, systems and methods

We require information from authors about some types of materials, experimental systems and methods used in many studies. Here, indicate whether each material, system or method listed is relevant to your study. If you are not sure if a list item applies to your research, read the appropriate section before selecting a response.

Materials & experimental systems

n/a	Involved in the study	
☐	☒	Antibodies
☐	☒	Eukaryotic cell lines
☒	☐	Palaeontology and archaeology
☒	☐	Animals and other organisms
☒	☐	Clinical data
☒	☐	Dual use research of concern
☒	☐	Plants

Methods

n/a	Involved in the study	
☒	☐	ChIP-seq
☒	☐	Flow cytometry
☒	☐	MRI-based neuroimaging

Antibodies

Antibodies used	anti-FLAG antibody (Agilent, Cat# 200474-21), anti β-actin (Cell Signaling, Cat# 4970S), HRP conjugated goat anti-rat immunoglobulin-G at 1:5000 dilution (Cell Signaling Technology, Cat# 7077S), HRP conjugated goat anti-rabbit immunoglobulin-G at 1:3000 dilution,

	Alexa-488 goat anti-rat antibodies at 1:200 (Thermo Fisher Scientific, Cat#A11006) (Cell Signaling Technology, Cat# 7074S)
Validation	The validation information for each antibody can be found on the manufacture's website.

Eukaryotic cell lines

Policy information about cell lines and Sex and Gender in Research

Cell line source(s)	HEK293 from ATCC
Authentication	Has been authorized by ATCC
Mycoplasma contamination	No Mycoplasma contamination
Commonly misidentified lines (See ICLAC register)	None
